# Rifabutin Use in *Staphylococcus* Biofilm Infections: A Case Series

**DOI:** 10.3390/antibiotics9060326

**Published:** 2020-06-13

**Authors:** James B. Doub, Emily L. Heil, Afua Ntem-Mensah, Renaldo Neeley, Patrick R. Ching

**Affiliations:** 1 Division of Infectious Diseases, University of Maryland School of Medicine, Baltimore, MD 21201, USA; rneeley@som.umaryland.edu; 2 Department of Pharmacy Practice and Science, University of Maryland School of Pharmacy, Baltimore, MD 21201, USA; eheil@rx.umaryland.edu; 3 Department of Infectious Disease, Faith Regional Health Services, Norfolk, NE 68701, USA; anmensah@frhs.org; 4 Department of Medicine, University of Maryland Medical Center Midtown Campus, Baltimore, MD 21201, USA; patrick.ching@umm.edu

**Keywords:** biofilms, rifabutin, *Staphylococcus*, drug interactions, periprosthetic joint infection

## Abstract

This is a case series of 10 patients who had staphylococcal biofilm infections that were treated with adjuvant rifabutin therapy instead of rifampin therapy. In these cases, rifampin was contraindicated secondary to drug–drug interactions with the patients’ chronic medications. Rifabutin therapy was well tolerated with no side effects. As well, no patients had recurrence of their staphylococcal infections. This case series shows that rifabutin can be a beneficial adjuvant therapy in *Staphylococcus* biofilm infections when drug–drug interactions limit the use of rifampin.

## 1. Introduction

Prosthetic joint replacements, vascular grafts, spinal fusions and other surgical interventions involving prosthetic material have improved the quality of life and reduced morbidity for a countless number of patients [1,2]. However, when these prosthetic materials become infected, we have limited therapeutic options to cure these infections [1,2]. Bacterial biofilms are the main reason these infections are so hard to cure with conventional antibiotics. Unfortunately, given the architecture and the low metabolic activity of bacteria in biofilms, conventional antibiotics have limited activity against bacteria in these sessile states [3]. In biofilms, bacteria can be up to 1000 times more resistant to antibiotics compared with the same bacteria in a planktonic state [3].

Approximately 50–60 percent of prosthetic joint infections (PJI) are caused by *Staphylococcus* species [4]. Rifampin is the most widely used anti-staphylococcal antibiotic that has in vitro activity to *Staphylococcus* biofilms [5,6,7]. Numerous guidelines recommend using rifampin as an adjuvant therapy to help cure staphylococcal biofilm infections [8,9]. Unfortunately rifampin is a strong inducer of multiple cytochrome P450 (CYP450) oxidative enzymes and the P-glycoprotein (P-gp) transport system. CYP450 enzymes play a role in the metabolism of many medications, and P450 enzyme inducers can increase the metabolism of medications that are CYP450 substrates. P-gp is extensively expressed in the intestinal epithelium and liver cells where drugs can be pumped back into the intestinal lumen or bile ducts, respectively. One study suggested that up to 30 percent of patients can have significant drug–drug interactions, not allowing for rifampin to be used [10]. Rifabutin has comparable in vitro anti-*Staphylococcus* activity to planktonic and biofilm states, but is a weaker inducer of fewer CYP450 enzymes and does not affect transport proteins such as P-gp [10]. Therefore, rifabutin has less drug–drug interaction potential and could be used in patients with multiple comorbidities that require numerous concomitant medications. Unfortunately, clinical data supporting its use for this indication are limited. In this case series, we present a group of patients with staphylococcal biofilm infections who were successfully treated with adjuvant rifabutin therapy instead of rifampin secondary to significant drug–drug interactions. To our knowledge, this is the first reported use of in vivo rifabutin therapy for staphylococcal biofilm infections.

## 2. Cases

Rifabutin was used instead of rifampin for 10 patients at the University of Maryland Medical Center over a three-year period. All patients had chronic staphylococcal infections involving prosthetic material; six with methicillin-sensitive *S. aureus* (MSSA), three with methicillin-resistant *S. aureus* (MRSA) and one with both MRSA and MSSA. The clinical isolates were all obtained from aerobic bacterial cultures of the prosthetic material at the time of surgical intervention. The *S. aureus* isolates all had minimal inhibitory concentrations for rifampin that were less than 0.5 mg/L. All patients received rifabutin instead of rifampin due to potential drug–drug interactions with the patients’ other concomitant medications including methadone (*n* = 3), anticoagulants (*n* = 5), anti-arrhythmic medications (*n* = 2), antipsychotic medications (*n* = 1) and anti-retroviral medications (*n* = 1)—some patients were on multiple medications that were contraindicated with rifampin. Rifabutin was used in combination with standard of care antibiotic therapy for at least six weeks. Standard of care antibiotics included cefazolin (*n* = 4), ceftaroline (*n* = 3), nafcillin (*n* = 1), daptomycin (*n* = 1) and levofloxacin (*n* = 1). All patients remained on rifabutin for the duration of their intravenous antibiotics as indicated in Table 1. No rifabutin therapy was continued longer than the intravenous antibiotic durations indicated.

Surgical intervention was performed on all patients. One-stage revision refers to the removal of prosthetic material and placement of new prosthetic material during the same surgery. Two stage revision refers to the removal of prosthetic material and placement of prosthesis after an intravenous antibiotic course. In this case series, all patients were followed for at least one year, and no patients experienced recurrence of infection. No patients experienced any rifabutin-attributed adverse events. Further details on patient cases can be found in Table 1.

## 3. Discussion

Rifamycins were discovered in the 1950s when they were isolated from *Amycolatopsis mediterranea* and were noted to have activity against *Mycobacterium tuberculosis* [11]. Since then, additional semisynthetic rifamycins have been created which include rifabutin, rifapentine and rifaximin. These antimicrobial agents have a broad range of activity against mycobacterium, Gram-positive bacteria and even some Gram-negative bacterial species: *Haemophilus*, *Neisseria* and others [11]. Rifamycins have excellent bactericidal staphylococcal activity [10]. Their mechanism of action is through inhibition of bacterial DNA-dependent RNA polymerase, which blocks the elongation of bacterial RNA [12]. This is independent of bacterial division, therefore resulting in activity against bacteria with low metabolic activity, similar to bacteria that are present in biofilms [13]. Rifamycins are also active in acidic, anaerobic environments and can accumulate in neutrophils and osteoblasts [14,15,16]. These properties allow for rifamycins to be attractive agents in staphylococcal biofilm infections [15]. However, when used alone, there is a high risk of the development of resistance [13,17]. As a consequence, rifamycins should not be used alone but rather as adjuvant therapies when treating staphylococcal biofilm infections. Resistance usually occurs from mutations of the *rpoB* gene [18]. Some studies suggest *rpoB* mutations confer cross-resistance to other rifamycins [18]. However, further in vitro studies are needed to evaluate if all *rpoB* mutations have cross-resistance to other rifamycins or if some retain activity.

The use of rifampin can be poorly tolerated given gastrointestinal intolerances and liver toxicity. As a strong inducer of multiple CYP450 enzymes including CYP3A4 and CYP2C19, it can lead to the increased metabolism of many medications that are CYP450 substrates (use CYP450 enzymes in their metabolic pathway) such as anticoagulants, HIV medications, anti-arrhythmics and methadone, to name a few. These drug–drug interactions can significantly limit its use. This case series displays cases where rifampin could not be used mainly secondary to interactions with anticoagulation medications. However, Table 1 shows additional patients in which rifampin could not be used because of significant drug–drug interactions with some other medications such as methadone, HIV medications and antipsychotics.

Compared with rifampin, rifabutin has the same anti-staphylococcal mechanism of action but it has a better toxicity profile and longer half-life [19]. Rifampin is associated with adverse reactions such as hepatitis, cholestasis, dermatological events and gastrointestinal disturbances. While rifabutin is not free from potential toxicity and can still be associated with hepatitis and uniquely uveitis, it is associated with fewer adverse events than rifampin [20,21]. In addition, rifabutin’s in vitro anti-*Staphylococcus* activity is comparable to rifampin [10]. It is also a weaker inducer of various CYP450 enzymes and does not induce or inhibit any transport proteins. This results in fewer drug–drug interactions compared with rifampin. This case series suggests that rifabutin is a viable option when rifampin is contraindicated in staphylococcal biofilm infections.

To this research team’s knowledge, this is the first published use of rifabutin in treating in vivo staphylococcal biofilm infections. Therefore, there are limited data to suggest the appropriate dosing of rifabutin in staphylococcal infections. Here at the University of Maryland Medical Center, we use an oral dose of 300 mg daily. This dose was adopted from rifabutin dosing used in mycobacterial and *H. pylori* infections. Lower doses could potentially be equally advantageous but the paucity of data with respect to rifabutin use in staphylococcal infections limits proper dosing regimens. This could be particularly advantageous as rifabutin at 150 mg per day has been shown to have the best risk–benefit ratio in efficacy and lowest incidence of adverse effects in tuberculosis treatment [21]. However, until further studies are conducted, we suggest a dose of 300 mg daily.

Rifabutin was well tolerated and no adverse events were observed. There were no cases of uveitis, but this case series is limited in its small sample size. Larger studies will need to be conducted to compare tolerability and toxicity to rifampin. In addition, this case series only had patients with *Staphylococcus aureus* (four MRSA and six MSSA) infections. No coagulase negative staphylococcal infections were studied in this case series. However, the in vivo activity of rifabutin to coagulase negative staphylococcal infections is likely comparable to rifampin, but limited data are available to support this claim [10,22].

## 4. Conclusions

In conclusion, in vitro data suggest that rifabutin has comparable activity to that of rifampin against staphylococcal planktonic and biofilm states [10]. Further studies could be conducted assessing the noninferiority of rifabutin compared with rifampin in staphylococcal biofilm infections, but these studies may require large sample sizes and funding for these may be arduous. Nonetheless, this case series reinforces the potential utility of using rifabutin in staphylococcal biofilm infections when rifampin is contraindicated.

## Figures and Tables

**Table 1:** Patients who received rifabutin for staphylococcal infection.

	Age	Sex	Infection	Reason for Rifabutin	Microbiology	Antimicrobial Therapy	Surgical Management	Adverse Effects	Outcome
1	35	F	vascular graft infection	methadone	MSSA	cefazolin for 6 weeks	one-stage revision	none	No recurrence at 2 years, no suppressive antibiotics
2	40	M	femoral hardware infection	anticoagulation and methadone	MRSA	ceftaroline for 6 weeks	hardware removal and intramedullary nail placement	none	No recurrence at 1 year, on suppressive antibiotics
3	42	M	hip prosthetic joint infection	anticoagulation	MSSA	cefazolin for 6 weeks	one-stage revision	none	No recurrence in 6 months, no suppressive antibiotics, lost to follow up
4	57	M	hip prosthetic joint infection	anticoagulation	MRSA	ceftaroline for 6 weeks	one-stage revision	none	No recurrence at 1 year, no suppressive antibiotics
5	58	M	knee prosthetic joint infection	antiarrhythmic	MRSA/MSSA	ceftaroline for 6 weeks	one-stage revision	none	No recurrence at 1 year, no suppressive antibiotics
6	62	M	femoral hardware infection	HIV medications	MRSA	daptomycin for 6 weeks	irrigation and debridement	none	No further MRSA infection at time of amputation
7	63	M	hip prosthetic joint infection	anticoagulation and antiarrhythmic	MSSA	cefazolin for 6 weeks	two-stage revision with cerclage wires retained	none	No recurrence at 2 years, no suppressive antibiotics
8	64	M	spinal hardware infection	methadone	MSSA	cefazolin for 6 weeks	irrigation and debridement	none	No recurrence, no suppression antibiotics
9	64	M	knee prosthetic joint infection	anticoagulation	MSSA	levofloxacin for 8 weeks *	two-stage revision	none	No recurrence at 2 years, no suppressive antibiotics
10	69	F	spinal hardware infection	antipsychotic	MSSA	nafcillin for 8 weeks	irrigation and debridement	none	No recurrence at 1 year, on suppressive antibiotics

MSSA—methicillin-sensitive *Staphylococcus aureus*, MRSA—methicillin-resistant *Staphylococcus aureus*. * This patient had severe allergies to penicillin, cephalosporins, vancomycin, daptomycin and pancytopenia with linezolid. Therefore, a unique regimen of levofloxacin and rifabutin was used for his MSSA PJI.
